# Supplementary material for: Glia-derived ATP inversely regulates excitability of pyramidal and CCK-positive neurons
Source: Nat Commun. 2017 Jan 27;8:13772. doi: 10.1038/ncomms13772 (PMC5290168; doi:10.1038/ncomms13772)
Supplement: Supplementary Information — Supplementary Figures, Supplementary Table, and Supplementary Methods. [file ncomms13772-s1.pdf]

Supplementary Figure 1

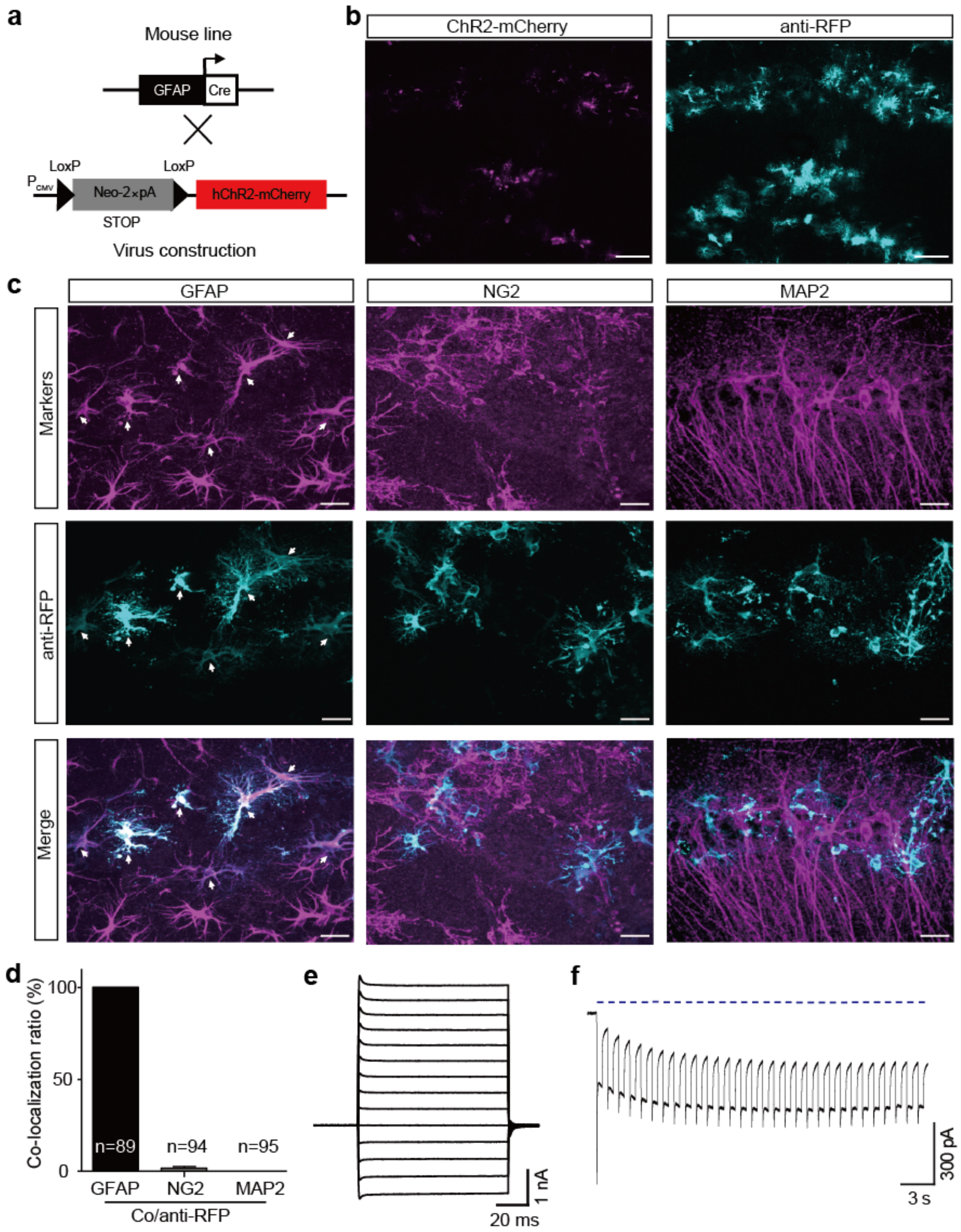

Supplementary Fig. 1. ChR2-mCherry is specifically expressed in astrocytes. (a) Schematic diagram showing adeno-loxP-STOP-loxP (LS_2L)-ChR2-mCherry with Cre-dependent expression of ChR2. Neo-2xpA, a neomycin STOP cassette with two polyadenylation sites. (b) Left: confocal image showing the expression level of ChR2-mCherry. Right: anti-RFP antibody staining to highlight the ChR2-expressing cells. Scale bars, 50 μm. (c) Expression of ChR2-mCherry (cyan) in the hippocampal CA1

region of GFAP-Cre mice and its co-localization (white arrows) with the astrocyte-specific marker GFAP (magenta, left), but not with the NG2 glial cell marker NG2 (magenta, middle) or the neuronal marker MAP2 (magenta, right). Scale bars, 50 μm. (d) Statistical results of C. Note that numbers indicate cells being analyzed. (e) Current responses from an astrocyte evoked by step voltages (100 ms, 20 mV) from –160 to +100 mV. (f) Light pulses (blue bars, 500 ms, 1 Hz) reliably induced inward currents in a ChR2-expressing astrocyte in a hippocampal slice.

Supplementary figure 2

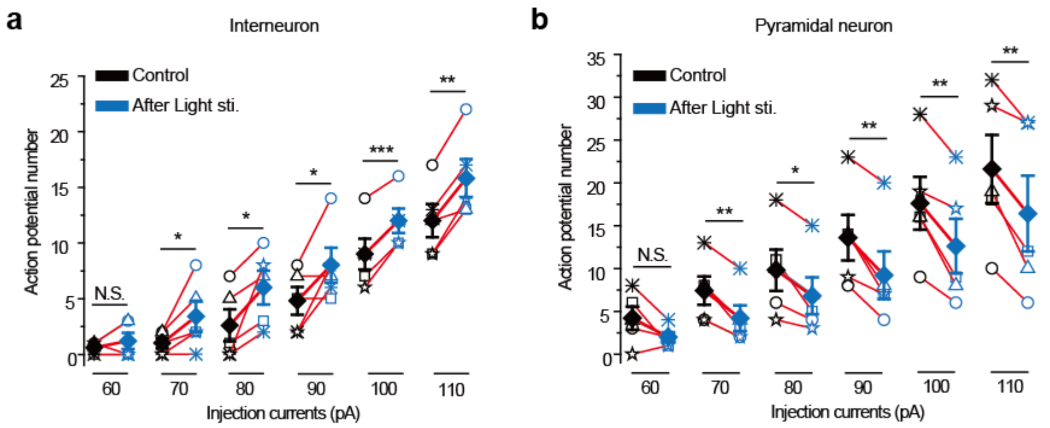

Supplementary Fig. 2. Summary of light-induced AP change in a step-current injection protocol. (a) Summary of change in interneuron AP numbers before (black) and after (blue) light stimulation at each current injection level. Open and filled symbols represent individual and averaged AP numbers, respectively. Paired Student's *t* test, 60 pA: P=0.187, 70 pA: P=0.04, 80 pA: P=0.0217, 90 pA: P=0.0389, 100 pA: P=0.00034, 110 pA: P=0.0033. (b) Summary of change in pyramidal neuron AP numbers before (black) and after (blue) light stimulation at each current injection level. Open and filled symbols represent individual and averaged AP numbers, respectively. Paired Student's *t* test, 60 pA: P=0.054, 70 pA: P=0.0026, 80 pA: P=0.0115, 90 pA: P=0.0045, 100 pA: P=0.0059, 110 pA: P=0.0054.

Supplementary figure 3

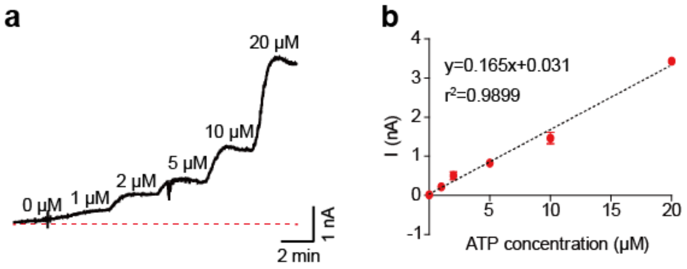

Supplementary Fig. 3. Standard curve of ATP-specific biosensor. (a) Representative trace showing current induced by various concentrations of ATP standard samples (from 1-20 μM). (b) Standard curve of an ATP-specific biosensor. Light stimuli induced increase of ATP concentration was calculated using the function obtained from linear fitting.

Supplementary figure 4

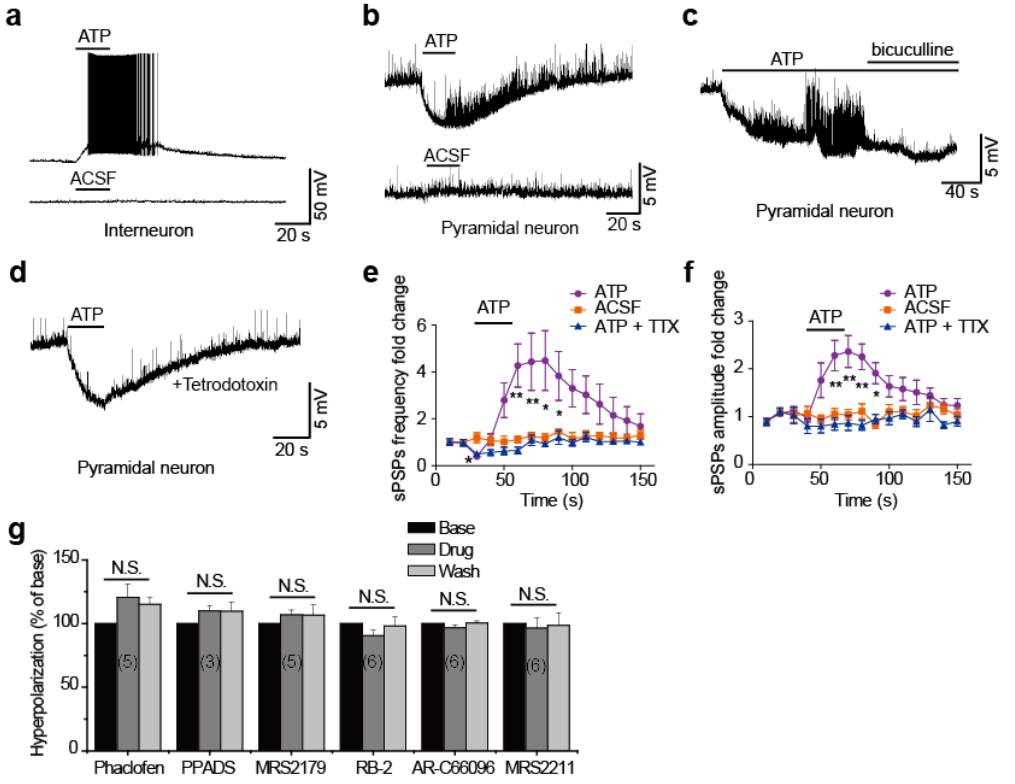

Supplementary Fig. 4. A high concentration of ATP induces robust depolarization in interneurons and hyperpolarization in pyramidal neurons. (a) Upper: representative trace of 100 μM ATP-induced depolarization in an interneuron. Note that such depolarization was sufficient to induce AP firing. Lower: HEPES-buffered aCSF puffing had no evident effect on membrane potential in the same neuron. (b) Upper: representative trace of 100 μM ATP-induced hyperpolarization in a pyramidal neuron. Note the transient inhibition followed by potentiation of spontaneous post-synaptic potentials (sPSPs) during ATP puffing. Lower: HEPES-buffered aCSF puffing did not induce an evident membrane potential change in the same neuron. (c) Example trace showing the increased frequency and amplitude of PSPs in a pyramidal neuron during ATP puffing was totally blocked by 10 μM bicuculline. (d) Representative trace showing the increased frequency and amplitude of PSPs in a pyramidal neuron during ATP puffing was totally blocked by 0.5 μM tetrodotoxin. (e, f) Statistical analysis of sPSPs frequency (e, two way ANOVA, $F_{(2, 315)}=70.22$, $P<0.0001$) and amplitude (f, two way ANOVA, $F_{(2, 315)}=57.72$, $P<0.0001$) changes in (b) and (d). (g) Summary data showing the percentage inhibition of ATP-induced hyperpolarization by various antagonists (Student's *t* test, phaclofen: P=0.144, PPADS: P=0.523, MRS2179: P=0.334, RB-2: P=0.0926, AR-C66096: P=0.0981, MRS2211: P=0.905). Data are normalized to the hyperpolarization amplitude induced by ATP in the absence of antagonists. N.S., not significant.

Supplementary figure 5

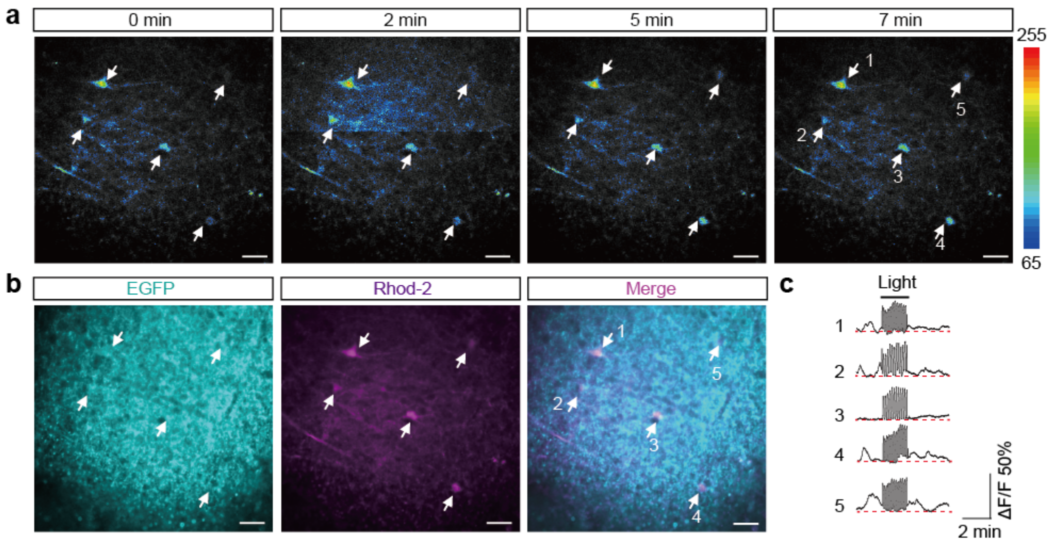

Supplementary Fig. 5. Light-stimuli have no effect on calcium signal in astrocytes expressing EGFP in brain slice. (a) Example time-lapse confocal imaging of Ca^{2+} signals. Light stimuli was applied at time point 2 min and lasted for 2 min. (b) Confocal images showing the EGFP-expressing astrocytes (cyan) loaded with the Ca^{2+} fluorescent dye Rhod-2 (magenta). Images are from the same field as in (a). Scale bars in (a) and (b) indicate 20 µm. (c) Example traces of light-induced Ca^{2+} signals (*ΔF/F*) in astrocyte as numbered in (a) and (b). Dotted lines indicate base level of Ca^{2+} signals.

Supplementary figure 6

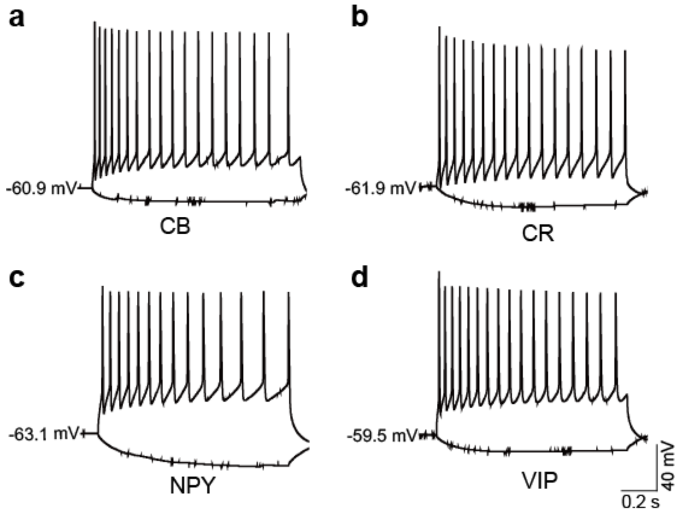

Supplementary Fig. 6. Sample traces showing the membrane potential change and AP discharge pattern of CB, CR, NPY, and VIP interneurons in response to 1 second 20 pA hyperpolarizing and 100 pA depolarizing pulse stimuli.

Supplementary figure 7

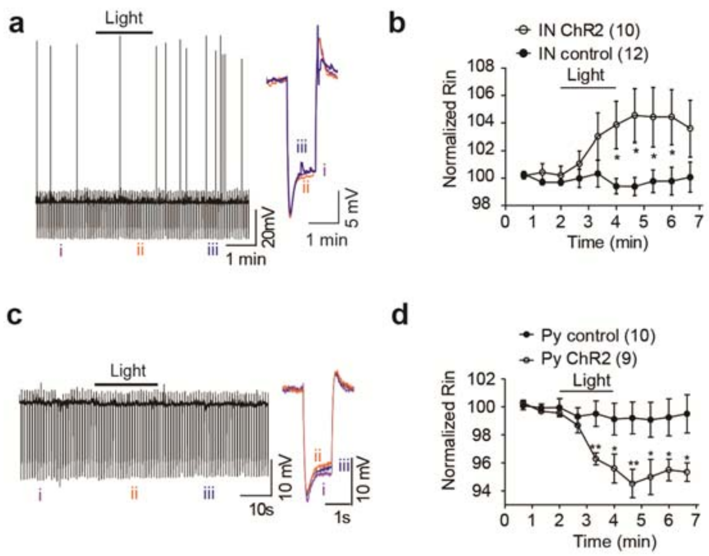

Supplementary Fig. 7. Light stimuli induced input resistance changes in interneuron and pyramidal neuron. (a) Sample trace showing interneuron input resistance (Rin) recorded before, during, and after light-stimuli monitored by -50 pA current injection every 5 s. (i), (ii), and (iii) indicate representative Rin traces in each period as indicated. (b) Time-course of the interneuron Rin change during light-stimuli in ChR2-expression slices (open circles) or EGFP expression slices (filled circles) (two way ANOVA, $F_{(1, 200)}$=28.34, P<0.0001). Data are normalized to the Rin averaged from 2 min of recording before light stimuli in each case. (c) Sample trace showing pyramidal neuron Rin recorded before, during, and after light stimuli monitored by -50 pA current injection every 5 s. (i), (ii), and (iii) indicate representative Rin traces in each period as indicated. (d) Time-course of interneuron Rin changes before, during and after light stimuli in ChR2-expression slices (open circles) or EGFP-expression slices (filed circles) (two way ANOVA, $F_{(1, 170)}$=39.45, P<0.0001). Data are normalized to the Rin averaged from 20 s of recording before drug application in each case.

Supplementary figure 8

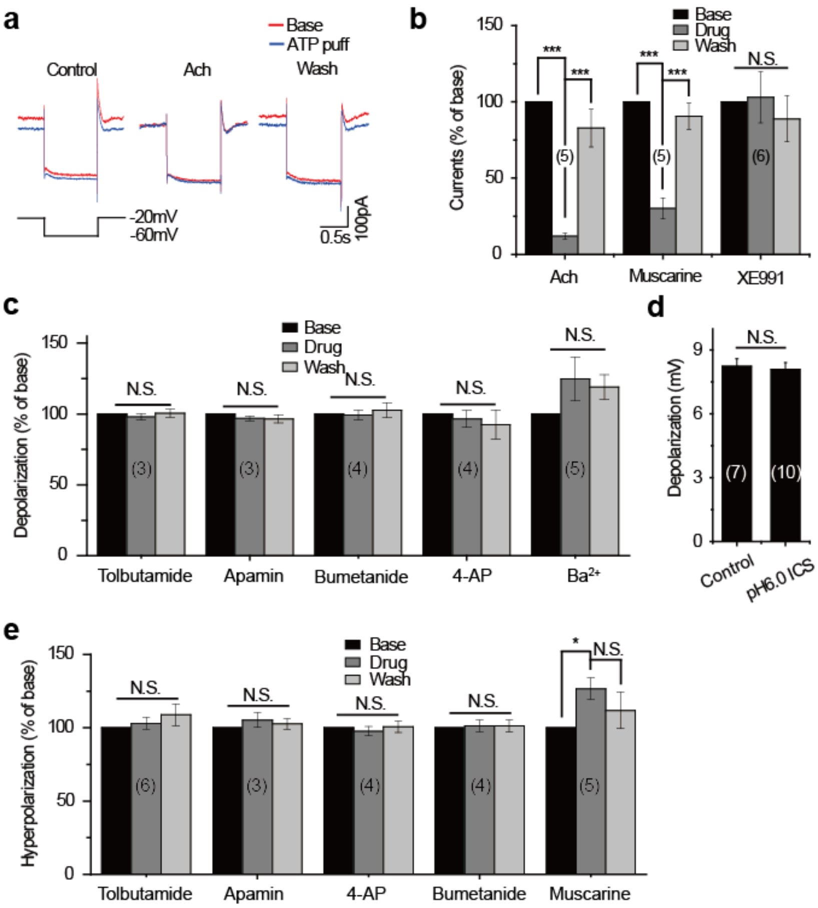

Supplementary Fig. 8. Involvement of K^+ channels in the ATP-induced modulation of neuronal excitability. (a) Representative traces showing the inhibition of ATP on standing outward K^+ current (I_{kso}), an effect that was unmasked by 0.5 mM acetylcholine (ACh). (b) Summary data showing the percentage of ATP-induced inhibition of I_{kso} in the presence of Ach (Student's *t* test, ***P<0.0001, ***P<0.0001), muscarine (Student's *t* test, ***P<0.0001, ***P<0.0001), and XE991 (Student's *t* test, P=0.628). Data are normalized to the current inhibition by ATP in the absence of antagonists. (c) Summary data showing the percentage inhibition of ATP-induced depolarization by various K^+ channel blockers (Student's *t* test, tolbutamide: P=0.628, apamin: P=0.772, bumetanide: P=0.818, 4-AP: P=0.788, Ba^{2+}: P=0.214). Data are normalized to the depolarization amplitude induced by ATP in the absence of blockers. (d) Summarized data showing the effect of intracellular acidification (pH 6.0, Student's t test, P=0.628)) on ATP-induced depolarization. (e) Summary data showing the percentage inhibition of ATP-induced hyperpolarization by various K^+ channel blockers (Student's *t* test, tolbutamide: P=0.956, apamin: P=0.611, 4-AP: P=0.326, bumetanide: P=0.636, muscarine: *P=0.024, P=0.064). Data are normalized to the hyperpolarization amplitude induced by ATP in the absence of blockers. N.S., not significant.

Supplementary figure 9

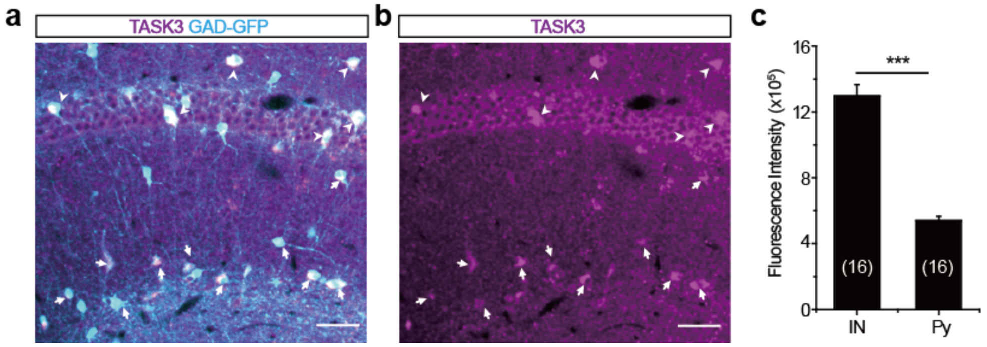

Supplementary Fig. 9. The expression levels of TASK3 differ in pyramidal neurons and interneurons. (a, b) Confocal images showing the expression level of TASK3 in pyramidal neurons and interneurons. Co-localization is indicated by arrows in the SR/SLM and by arrowheads in the SO/SP. Scale bars, 60 µm. (c) Summary of TASK3 expression level in pyramidal neurons and interneurons as reflected by fluorescence intensity (Student's *t* test, ***P <0.0001).

Supplementary table 1. Sequences of PCR primers.

Gene	GenBank no.	RT-PCR primers	Outer primers	Size	Inner primers	Size
P2Y1	NM_008772.4	1670*: ATTACTCGTGGCT-	Sence, 857: CGTGGCTATCTGGATGTTCGT	707	Sence, 1054: ACGGTAGCATCTTGTTCCTCA	178
		CGGGACAGTCTCCTTCT	Antisence, 1563: AAGTGGCATAAACCCTGTCGT		Antisence, 1231: CCGAGTCCCAGTGCCAGAGTA	
A1	NM_001008533.2	2155: GTGCGAAGGCTGA-	Sence, 1358: CTGGCTCTGCTTGCTATTGCTG	643	Sence, 1362: CTCTGCTTGCTATTGCTGTG	276
(Adora1)		GGAGGAACAGTGGGACA	Antisence, 2000: GGCATCGGAAGTGGTCGTT		Antisence, 1637: CCCAGACGAAGAAGTTGAA	
TASK-3	NM_001033876.1	829: GCGACGCACGGAC-	Sence, 246: TTCCTTCTACTTCGCCATCA	544	Sence, 248: CCTTCTACTTCGCCATCACT	137
(KCNK9)		AGACACTCTTCTTGGATTT	Antisence, 789: AATCTCCGCAACTTCTCCC		Antisence, 384: CAGGCTCTGGAACATAACCA	
GAD 65	NM_008078.2	1545: CGGTTAGGGGACA-	Sence, 1049: TCTTTTCTCCTGGTGGTGCC	391	Sence,1159: GCGTTCACATCAGAGCATAG	218
(Gad2)		CCCATCATCTTGTGAGGA	Antisence, 1439: CCCCAAGCAGCATCCACAT		Antisence, 1376: AGAGGATCAAAAGCCCCATA	
CAMK II	NM_009792.3	1036: CTCGGGTACATAGGT-	Sence, 379: GCGGAGGAAACAAGAAGAAC	648	Sence, 700: TCCTGAACCCTCACATCCAC	151
		GGCAATGGTAGGGTGATC	Antisence, 1026: TAGGTGGCAATGGTAGGGTG		Antisence, 850: ATCTGCCATTTGCCGTCC	
CCK	NM_031161.3	666: CGTCCATAGCATAGCAA-	Sence, 110: TGTCTGTGCGTGGTGATGG	548	Sence, 288: ACATCCAGCAGGTCCGCAA	234
		CATTAGGTCTGGGAGTCA	Antisence, 657: CATAGCAACATTAGGTCTGGGAG		Antisence, 521: AGACATTAGAGGCGAGGGGT	
CB	NM_009788.4	2448: TCACCTTCGGTCTAA-	Sence, 1518: GGCTTGGTAAGGGAAGGTAG	555	Sence, 1597: GAGAGTATGACCATAGCCCATT	198
(Calb1)		AGTCACTGCTTCCAAATACG	Antisence, 2072: ATGTGGGTCAGTGAAGGTTT		Antisence, 1794: AGCAGATACCCTTGGTGGAA	
CR	NM_007586.1	1094: TCAATCGTGACCC-	Sence, 154: GATGCTGACGGAAATGGGTA	585	Sence, 540: GACCATACTACGGATGTTTGACTT	168
(Calb2)		AACGCAGGCACAACT	Antisence, 738: GAGGGCGTCCAGTTCATTC		Antisence, 707: CCGCTTCCATCCTTGTCA	
NPY	NM_023456.2	320: CGCTGGCCTTCATTAA-	Sence, 89: CGAATGGGGCTGTGTGGA	294	Sence, 112: CCTCGCTCTATCTCTGCTCGT	218
		GAGGTCTGAAATCAGTGTCT	Antisence, 382: AAGTTTCATTTCCCATCACCACAT		Antisence, 329: CGTTTTCTGTGCTTTCCTTCA	
VIP	NM_011702.2	1287: GTGATGCGTCTCTG-	Sence, 52: CGGGAACAGACTGGTGGAG	391	Sence, 170: GCCAGAAGCAAGCCTCAGTT	121
		AAGTAGCCTTTGGGGATTG	Antisence, 442: TCTGCTGTAATCGCTGGTGA		Antisence, 290: GCATCCTGTCATCCAGCCTAC	

Note: Instead of using poly dT, we used gene-specific antisence primers with several modifies at 5' end to do the RT-PCR.

The name of the gene is listed in parentheses when it differs from the usual name of the marker.

*: Number indicates first base of the start codon.

Supplementary Materials and Methods

Electrophysiology

To assay neuronal excitability in current-clamp mode, depolarizing current (50-100 pA) was injected to maintain continuous AP firing at 0.5-1.5 Hz. Bicuculline (10 μM) and kynurenic acid (0.5 mM) were added to the perfusate to exclude the influence of synaptic transmission. Blue light or the indicated drugs were applied after at least 2 min of steady firing was recorded. All the light stimulation experiments were performed at 32°C to facilitate the release of ATP from astrocytes.

The resting membrane potential was held to approximately -60 mV for interneurons and -70 mV for pyramidal neurons when assessing the effects of exogenous ATP in current clamp mode, to minimize the impact of membrane potential on ATP-induced potential change. Exogenous ATP or ATPγs was diluted in HEPES-buffered aCSF with pH adjusted to 7.35 and puffed with a Picospritzer III (Parker Hannifin Corp.) for 20 s each time. We found no evident difference between ATP and ATPγs application, so these data were pooled. In some cases, 0.5 μM tetrodotoxin was added to the perfusate to block APs and spontaneous postsynaptic potentials (sPSPs) and reveal the underlying ATP-induced change in membrane potential. Standing outward K^+ current (Ik_{so}) was pre-activated by holding the membrane potential at -20 mV, and the deactivation current was recorded by stepping to -60 mV for 1 s every 10 s. In all the whole-cell electrophysiological experiments, accessory resistance was monitored before and after recording, and those with a >20% change were excluded from the analysis.

Slice culture

Brains from P13 C57/BL6 mice were cut in aCSF on a vibratome as above. Hippocampi were separated from slices and rinsed 3 times in complete Hanks' balanced salt solution (HBSS) containing (in mM): 2.5 HEPES, 30 glucose, 1 $CaCl_2$, 1 $MgSO_4$, 4 $NaHCO_3$, and 100 U penicillin-streptomycin (pH 7.4). After that, hippocampi were transferred to the surface of a Millicell-CM culture insert (Millipore) which was placed in a dish containing slice culture medium: 50% minimum essential medium, 25% heat-inactivated normal horse serum, 25% HBSS, and 0.65% glucose. In some cases, pertussis toxin (2 μg/ml) was added. After treatment for >24 h, hippocampi were used for electrophysiological recording.
